# Performance of a rule-based semi-automated method to optimize chart abstraction for surveillance imaging among patients treated for non-small cell lung cancer

**DOI:** 10.1186/s12911-022-01863-0

**Published:** 2022-06-03

**Authors:** Catherine Byrd, Ureka Ajawara, Ryan Laundry, John Radin, Prasha Bhandari, Ann Leung, Summer Han, Stephen M. Asch, Steven Zeliadt, Alex H. S. Harris, Leah Backhus

**Affiliations:** 1 Department of Cardiothoracic Surgery, Stanford University School of Medicine, 300 Pasteur Dr., Falk Research Building, Stanford, CA 94305 USA; 2 Department of Veterans Affairs, VA Palo Alto Healthcare System, Health Services Research and Development, Palo Alto, USA; 3 Department of Veterans Affairs, VA Puget Sound Healthcare System, Health Services Research and Development, Seattle, USA; 4 VA Information Resource Center (VIReC), Health Services Research and Development Service, Hines, USA; 5 Department of Radiology, Stanford University School of Medicine, Stanford, USA; 6 Quantitative Science Unit, Stanford University School of Medicine, Stanford, USA; 7 Department of Surgery, Stanford University School of Medicine, Stanford, USA

**Keywords:** Natural language processing, Lung neoplasms, Chart abstraction, Non-small cell lung carcinoma, Imaging surveillance

## Abstract

**Background:**

We aim to develop and test performance of a semi-automated method (computerized query combined with manual review) for chart abstraction in the identification and characterization of surveillance radiology imaging for post-treatment non-small cell lung cancer patients.

**Methods:**

A gold standard dataset consisting of 3011 radiology reports from 361 lung cancer patients treated at the Veterans Health Administration from 2008 to 2016 was manually created by an abstractor coding image type, image indication, and image findings. Computerized queries using a text search tool were performed to code reports. The primary endpoint of query performance was evaluated by sensitivity, positive predictive value (PPV), and F1 score. The secondary endpoint of efficiency compared semi-automated abstraction time to manual abstraction time using a separate dataset and the Wilcoxon rank-sum test.

**Results:**

Query for image type demonstrated the highest sensitivity of 85%, PPV 95%, and F1 score 0.90. Query for image indication demonstrated sensitivity 72%, PPV 70%, and F1 score 0.71. The image findings queries ranged from sensitivity 75–85%, PPV 23–25%, and F1 score 0.36–0.37. Semi-automated abstraction with our best performing query (image type) improved abstraction times by 68% per patient compared to manual abstraction alone (from median 21.5 min (interquartile range 16.0) to 6.9 min (interquartile range 9.5), p < 0.005).

**Conclusions:**

Semi-automated abstraction using the best performing query of image type improved abstraction efficiency while preserving data accuracy. The computerized query acts as a pre-processing tool for manual abstraction by restricting effort to relevant images. Determining image indication and findings requires the addition of manual review for a semi-automatic abstraction approach in order to ensure data accuracy.

**Supplementary Information:**

The online version contains supplementary material available at 10.1186/s12911-022-01863-0.

## Background

Lung cancer represents the leading cause of cancer deaths within the US [1].

Patients undergoing treatment with curative intent are routinely followed for ongoing cancer surveillance as the risk of recurrent lung cancer is estimated at 2–14% per patient-year and the risk of second primary lung cancer is 1–4% per patient year [2–4]. Data to inform recommendations on surveillance timing hinges on the ability of researchers to accurately categorize imaging tests as to their indications and findings. Such detailed data have historically required manual chart abstraction as the gold standard for capturing clinical events [5–7]. However, manual chart abstraction in clinical research is labor intensive and time consuming [8], thereby limiting the size of data sets that can be studied to inform practice. Thus, the majority of data regarding surveillance imaging following lung cancer treatment has been limited to small case series [9–15]. Consequently, guidelines regarding post treatment lung cancer surveillance from the National Comprehensive Cancer Network (NCCN) and other international entities are based on low level evidence and the opinions of expert panels of providers [16].

Other studies have been conducted using larger data sources in an attempt to inform surveillance guideline recommendations (e.g. Surveillance, Epidemiology and End Results Program (SEER) or Veterans Affairs Central Cancer Registry (VACCR)) [17, 18]. These administrative and claims-based datasets provide large amounts of data for population studies, providing sufficient power for robust analysis. They are limited, however, in that they often lack the clinical granularity required to fully inform decision making [19–21]. Pairing this data with the gold standard method of obtaining clinical data—chart abstraction—would provide a significant amount of relevant clinical information to truly understand current practices of post treatment surveillance but is often impractical due to the expense and labor of such abstraction.

To address this problem, some have advocated for automating the process of chart abstraction through the use of machine-learning and natural language processing, thereby reducing the time burden and increasing efficiency. While these techniques have shown some promise overall, reliable methods to distinguish the indications for imaging studies in the context of lung cancer do not exist. Additionally, use of natural language processing suffers from limitations in accuracy [22]. At least one prior study compared abstraction of lung cancer imaging reports using natural language processing alone, manual abstraction alone, and a combination of the two methods and found the combination of manual abstraction and natural language processing to be the most accurate in identifying findings suspicious for lung cancer. However, this study only examined CT chest reports and did not specifically focus on post-treatment surveillance imaging which can be more complex studies to interpret by radiologists [23]. Another study also examined CT chest imaging reports using a rule-based natural language processing algorithm to identify lung nodules and more complex machine learning algorithms to determine the presence of concerning features. However, again, the authors only examined one type of imaging modality and did not focus on post-treatment surveillance imaging [24]. Though CT chest is recommended by NCCN guidelines for post-treatment surveillance [16], in clinical practice, multiple different imaging types are utilized routinely to rule out or diagnose recurrence in these patients.

### Objective

We sought to develop a semi-automated approach to chart abstraction to speed identification and evaluation of post treatment surveillance imaging and to test the efficiency and accuracy of this method. The method combines the advantages of a large administrative dataset with important clinical details to produce a rich data source for robust analyses. This is a part of a larger study of a national cohort of lung cancer patients within the Veterans Health Administration (VHA) examining data from the Corporate Data Warehouse (CDW), that includes full clinical and radiographic notes and reports as unstructured elements. The methods herein describe the use of the Veterans Indexed Search for Analytics (VISA), for improved time efficiency as well as its limitations by defining the point at which manual chart abstraction must supplement VISA use to produce accurate results.

In this study, we hypothesized that VISA queries will function at an intermediate level in terms of clinical accuracy. We anticipated they would provide insufficient search results (mid-level sensitivity and positive predictive value) to appropriately characterize the clinical indication for the radiology study and the image findings on their own. However, when combined with manual abstraction (the semi-automated method), VISA query use (specifically using the image type query) will result in an increase in efficiency of abstraction as compared to manual abstraction alone.

## Methods

### Cohort creation

This study was evaluated by the joint Stanford and Veterans Administration IRB and waivers of consent were granted. The larger study population includes Veterans who had been diagnosed with non-small cell lung cancer (NSCLC) between 2008 and 2016 that were present in the Veterans Administration (VA)/CDW database. This data source combines structured administrative data elements (e.g. International Classification of Diseases (ICD) and procedure codes) from multiple sources into a central resource [7, 25].

We included Veterans with a relevant ICD-9 diagnosis of lung cancer that underwent treatment with curative intent, defined by relevant treatment and procedure Current Procedural Terminology (CPT)/Healthcare Common Procedure Coding System (HCPCS) codes (lung resection, chemotherapy and radiotherapy or combination thereof) (Additional file 1: Appendix A: ICD-9 and CPT/HCPCS codes). Treatments and procedures were included if they occurred between one month before and 6 months after diagnosis. Patients were excluded if they died within 6 months of diagnosis, had any previous cancer within the prior 5 years, or had stage IV disease. Following exclusion, 17,472 total Veterans were included in the final cohort from the initial 185,112 patients. (Additional file 1: Appendix B: Consort diagram).

### Radiographic index preparation

A searchable radiology index was created which housed the full text radiology reports within the aforementioned timeframe for all patients in this cohort. The radiology reports were saved in a Text Integration Utility (TIU) format in the CDW. Relevant CDW radiology note domains (patient indentifier (PatientICN), radiology exam, radiology report text, date of report, etc.) were queried using SQL to identify all radiology related text documents. Separately, relevant structured clinical variables were queried from the CDW (PatientICN, date of diagnosis, cancer histology, stage, etc.). All retrieved information was then stored in a relational database. The Lucene-based VISA tool (developed in part by members of our team) indexed the aforementioned radiology texts stored in the relational database and linked them with the structured clinical variables via the patient identifier combining the two data sources [26].

### Lucene-based search tool development

To characterize all imaging performed during the post-treatment period, we defined our main outcome variables as image type, image indication, and image findings (the latter as described by the reporting radiologist). VISA, a Lucene-based open-source full text search engine written in Java, was used to index the radiology reports, search the radiology reports for text relevant to the user’s query, and then to abstract radiology data from the reports. This tool was developed in part by members of the team and has been previously applied to clinical provider documents within the medical chart to capture conversations surrounding the initiation of dialysis for chronic kidney disease and identify adverse childhood events in the Veteran population [6, 27]. The Lucene-based VISA text search tool, as described above, rapidly reviews the full text radiology reports within the index. Based on a user’s query, VISA returns the user-defined relevant full text radiology reports (organized by patient identifier and date of report) with highlighted relevant text snippets. In other words, a complex combination of key words can be automatically searched for, allowing for more efficient chart abstraction.

**Fig. 1:**
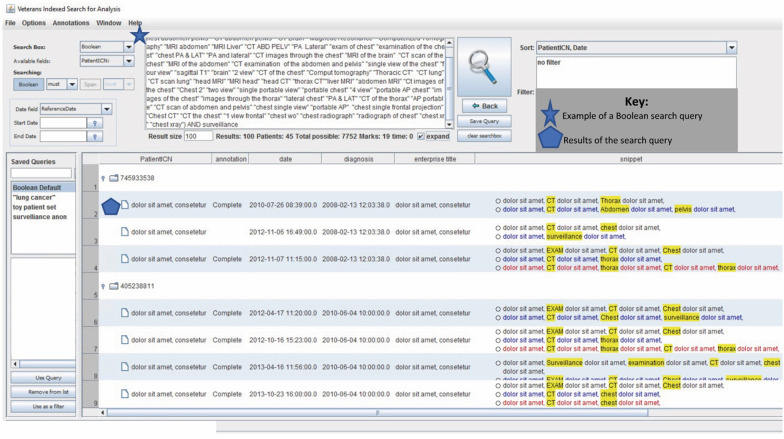
Veterans indexed search for analytics (VISA) tool. This figure includes a nonsense example of the appearance of the Veterans Indexed Search for Analytics (VISA) Tool. The star highlights an example of a Boolean search query. The subsequent results and snippets of text information with highlighted terms from the search query are seen below as represented by the pentagon

The VISA tool allows radiology reports to either be flagged and coded without manual review (computerized query only) or to be manually reviewed and annotated by a clinical abstractor (as in the case of our semi-automated review) (Fig. 1: VISA Tool). Three search query types are available for use in VISA—Boolean, Span, and Phrase Queries. Boolean queries allow system users to combine keywords with operators such as “AND” and “OR” to search the entire scope of the document to find relevant reports. Span queries retrieve documents where identified keywords are all present within a specified number of words. The “NOT” operator can be used in the span query. If keywords are linked by “NOT” within the aforementioned specified number of words, the document is not a match and is excluded. Phrase queries allow for a set of adjacent words within quotations to be found within a document. The VISA tool returns relevant report information including a patient identifying number (PatientICN), if the report has been abstracted by the reviewer already, the date of cancer diagnosis, the title of the document (enterprise title), and a snippet of the report text containing the highlighted queried terms.

**Fig. 2:**
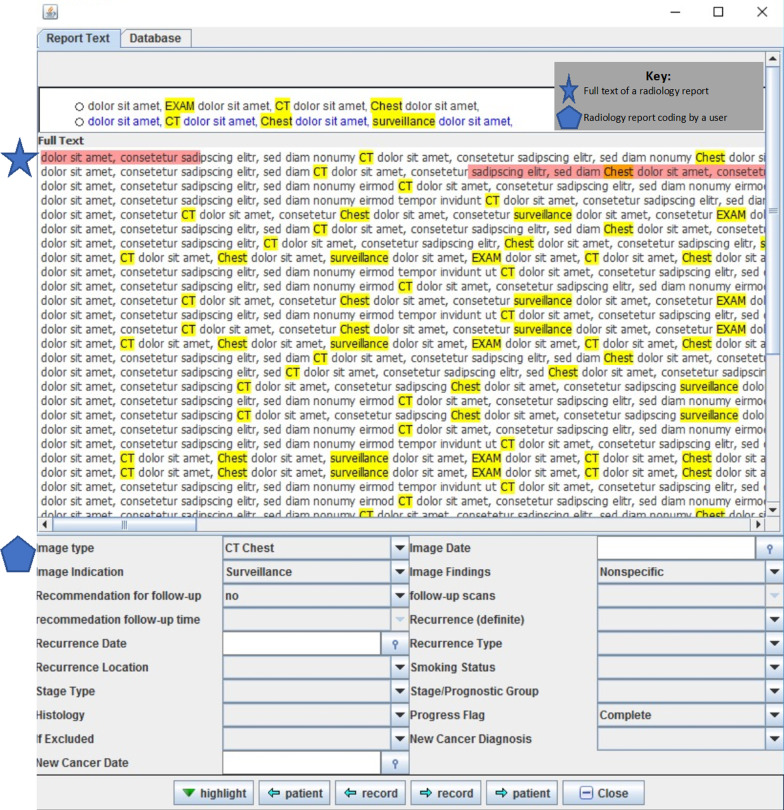
VISA data collection instrument. This figure includes a nonsense example of the appearance of the Veterans Indexed Search for Analytics (VISA) Data Collection Instrument tool. The star demarcates the full text of a radiology report. User highlighted text is in pink. Yellow highlighted text represents computer identified queried words and phrases. A pentagon represents an example of the way in which the radiology report may be coded by a user

VISA also houses the data collection instrument specific to our project. Thus, each radiology report can be reviewed by a clinical abstractor to code image type (CT chest, Chest x-ray, PET/CT, etc.), image indication (surveillance, symptoms, follow up from abnormal prior imaging, etc.), image findings (benign, suspicious for recurrence, definitive recurrence, etc.) and other relevant clinical variables (Fig. 2: VISA Data Collection Instrument). At the completion of abstraction, data are subsequently exported to a SQL based server.

### Manual abstraction training test performance

Clinical abstractors were trained to manually abstract radiology reports using a series of validated test data. Abstractors underwent three rounds of training using sets of 20–40 randomly selected reports from the 17,472 patient-cohort. They were required to reach a concordance rate of 95%. After the completion of manual abstraction training, abstractors timed themselves to determine the amount of time required to manually abstract data from patient reports.The time to abstract each patient’s set of reports over the course of a workday (8 h) was timed using stopwatches. The first time recorded the duration of time to abstract an entire patient chart (patient-level timing). This was defined by the time between opening a patient record and end of data abstraction for that patient. The second time recorded the duration of time to abstract each radiology report (report-level timing). This was defined by the time from opening each image report until after that report had been completely coded. Prior to the development of queries, the time to manually abstract the reports/ patient data as described above was recordered and stored. This manually abstracted set of patients would later be compared with a subsequent dataset that utilized semi-automated abstraction to determine the efficiency of each technique.

### Search query development

Queries were then developed to determine test performance of the VISA tool for our main outcomes of interest. A total of 4 queries were developed (Additional file 1: Appendix C: Search Queries). The goal of the first query was to identify the appropriate image types relevant to identifying lung cancer recurrence, metastasis, or second primary lung cancers and to exclude non-relevant imaging. Relevant image types included: CT chest, CT head, CT abdomen/pelvis, CT chest/abdomen/pelvis, chest x-ray, bone scan, PET/CT, MRI brain, and MRI body. Search terms and phrase queries were developed in an iterative process with the goal of capturing as many relevant studies as possible. The phrase queries were added into a Boolean search query with “OR” as the default operator between phrases. All subsequent searches were thus restricted to relevant imaging studies.

The second query sought to identify the radiology studies obtained specifically for the indication of post-treatment “surveillance”. This was selected as it was most relevant to the questions of the larger study to determine the ideal surveillance for asymptomatic patients following treatment for lung cancer. As per the first query, phrases associated with surveillance were identified and added into a Boolean search query with default operator “OR” between terms/phrases (Additional file 1: Appendix C: Search Queries). The last two queries sought to characterize imaging findings and more specifically, to identify studies with findings “suspicious” for recurrence, metastasis, or second primary lung malignancy and those where the radiologist indicated findings definitive for cancer recurrence, metastasis, or second primary lung cancer. Similar to “surveillance”, we limited the findings query to these two categories in order to focus on the variables that carried the most relevance for the larger study of surveillance and clinical outcomes. For the “suspicious” query, span queries were built that searched for combinations of terms signifying suspicious (e.g. “worrisome”, “concerning,”) and words that represented recurrence, metastatic disease, or second primary lung cancer within a 10-word span. If the words “not” or “no” were present within the 10-word span, those radiology reports were excluded. The “recurrence” query was built using words that indicated definitive cancer recurrence, metastatic disease, or second primary lung cancer within a 10-word span. If the words “not” or “no” were present within the 10-word span, those image reports were excluded (Additional file 1: Appendix C: Search Queries).

### VISA test performance for computerized queries

To test the computerized VISA tool test performance in coding radiology reports, we created a gold standard of completely manually abstracted data derived from a random sample of the parent study cohort of 17,472 patients. This gold standard cohort consisted of 361 patients with abstraction and annotations performed manually (Table 1: Characteristics of Gold Standard Manually Abstracted Radiology Reports). This cohort was used as a reference standard for test performance of the VISA tool computerized queries.

**Table 1:** Characteristics of gold standard manually abstracted radiology reports^α^

Characteristics of radiology reports	Annotated reports (N (%))
Image types	n = 3011
Bone scan	23 (0.8)
Chest X-ray	1320 (43.8)
CT abdomen/pelvis	127 (4.2)
CT chest	902 (30.0)
CT chest/abdomen/pelvis	123 (4.1)
CT head	149 (4.9)
MRI body	58 (1.9)
MRI brain	42 (1.4)
PET scan	267 (8.9)
Image indication	n = 3009
Surveillance	954 (31.7)
Symptomatic	649 (21.6)
Follow up from prior abnormal chest imaging	244 (8.1)
Follow up from prior abnormal other imaging	30 (1.0)
Other	480 (16.0)
Unknown	652 (21.7)
Image findings	n = 3008
Suspicious	331 (11.0)
Recurrence	110 (3.7)
Benign	959 (31.9)
Nonspecific	1019 (33.9)
Second primary lung cancer	14 (0.5)
Second primary cancer, other	11 (0.4)
Other (unrelated to cancer)	564 (18.8)

^α^The total number of reportss with image type annotated = 3011, the total number of reports with image indication annotated = 3009, the total number of reports with image findings annotated = 3008. 180 reports were found in our manually abstracted dataset that were coded as null, indicating that they were not relevant images. Thus the total number of reports representing 361 patients was 3191

We evaluated the performance of each computerized query applied (image type, image indication, and image findings) to our manually abstracted gold standard dataset using the VISA search tool. Sensitivity (recall), PPV (precision), F1 score, and specificity were calculated to evaluate performance of each query to accurately retrieve and characterize reports. All statistical analyses were performed using R [28].

VISA allows users to develop queries to search for reports that correspond to a specific annotation. The sensitivity of the query is thus the fraction of appropriately retrieved reports (true positives) out of all reports with that specific annotation (true positives + false negatives) in the gold standard cohort. The PPV of the query is the fraction of reports that are appropriately retrieved (true positives) out of all the retrieved reports (true positives + false positives). The F1 score is a summary statistic representing the harmonic mean of sensitivity and PPV, taking into account both false positives and false negatives. The specificity of a query is the fraction of reports that the lucene tool appropriately excluded (true negatives) out of all reports not matching a specific annotation (true negatives + false positives). A high-performing, efficient query has both high sensitivity (retrieves a majority of the relevant results) and high PPV (of the reports retrieved, a majority of them are relevant). A low PPV indicates that the query retrieved several irrelevant reports that must be reviewed manually for exclusion. A low sensitivity indicates that the query did not appropriately identify relevant reports. In those cases, manual abstraction is necessary for accuracy. A high specificity is also helpful as it indicates that this query excludes mostly irrelevant reports, though literature in this work tends to focus soley on the sensitivity and PPV of a search query [6, 29, 30].

### Semi-automated abstraction methods and timing

We determined the highest performing computerized queries (high sensitivity, PPV, and F1 score) and then combined this with manual abstraction for more accurate coding. First, the computerized query was applied to yield snippets of text with highlighted terms that were then viewed within the context of the larger report by the trained abstractor (Fig. 1). The abstractor had full access to all other reports for a given patient as well as the VISA database tab which included important clinically relevant data (date of diagnosis, histology, etc.) to assist with accurate coding as in the case of the full manual abstraction process. The abstractor would then apply the relevant data codes to each imaging report providing the most accurate accounting of the data based on the displayed text and snippet.

To determine the effect of semi-automated abstraction using VISA queries on efficiency of data abstraction, we compared the time required for semi-automated and manual abstraction at the patient-level and report-level. A new cohort of patients was abstracted to measure semi-automated abstraction times while the manual abstraction timing data was derived from patients in the gold standard manually abstracted cohort. For this comparison, we limited the use of VISA to the image type query given its superior performance as compared to the other queries (see “results” below). Using the same method described above, the time to abstract each patient’s set of reports using the semi-automated method over the course of an 8 h workday was timed using stopwatches. Time per patient (minutes), time per report (seconds), and number of reports per patient were recorded. Collected manual abstraction times described in the “Manual abstraction training test performance” section derived from the gold standard patient cohort were then compared with the semi-automated abstraction times using the Wilcoxon rank-sum test for non-parametric data for comparison of independent groups data. The number of reports per patient were compared using a simple t-test. Percent reduction in time from semi-automated abstraction to manual abstraction was also calculated.

## Results

The results of the performance of the image type query on the 361-gold standard manually abstracted patients are presented in Table 2. The image type query performed relatively well. Sensitivity was 85%, PPV 95%, F1 score 0.90 with respect to the query’s ability to retrieve relevant imaging reports. The query’s sensitivity to detect certain image types was also evaluated and is presented in Table 3. Sensitivities ranged from 79 to 100% for the image types of bone scan, chest X-ray, CT chest, CT chest/abdomen/pelvis, CT head, MRI brain, and PET scan. Sensitivity of the query was low for CT abdomen/pelvis (50%) and MRI body (48%). Performance of the “surveillance” indication query and image findings queries are presented in Table 2. The query for “surveillance” indication demonstrated sensitivity 72%, PPV 70%, and F1 Score 0.71. For study findings, the “suspicious” query demonstrated sensitivity 75%, PPV 25%, and F1 Score 0.37 and the “recurrence” query demonstrated sensitivity 85%, PPV 23%, and F1 Score 0.36.

**Table 2:** Overall performance of queries in the 361-patient manually abstracted cohort

Image annotation				
Image type: any relevant image			Gold standard manual abstraction	
			Relevant study	Not relevant study
	Automated Lucene tool result	Relevant study	2548	133
		Not relevant study	463	47
	Sensitivity^β^ (95% CI)	Specificity^γ^ (95% CI)	PPV^δ^ (95% CI)	F1 score^ε^
	85% (83–86%)	26% (20–33%)	95% (94–96%)	0.90
Indication: surveillance			Gold standard manual abstraction	
			Surveillance study	Not surveillance study
	Automated Lucene tool result	Surveillance study	690	292
		Not surveillance study	264	1763
	Sensitivity (95% CI)	Specificity (95% CI)	PPV (95% CI)	F1 score
	72% (69–75%)	86% (84–87%)	70% (67–73%)	0.71
Finding: suspicious			Gold standard manual abstraction	
			Suspicious finding on study	No suspicious finding on study
	Automated Lucene tool result	Suspicious finding on study	247	755
		No suspicious finding on study	84	1922
	Sensitivity (95% CI)	Specificity (95% CI)	PPV (95% CI)	F1 score
	75% (70–79%)	72% (70–73%)	25% (22–27%)	0.37
Finding: recurrence only			Gold standard manual abstraction	
			Recurrence on study	No recurrence on study
	Automated Lucene tool result	Recurrence on study	105	353
		No recurrence on study	19	2531
	Sensitivity (95% CI)	Specificity (95% CI)	PPV (95% CI)	F1 score
	85% (77–91%)	88% (87–89%)	23% (19–27%)	0.36

^β^Sensitivity = true positive/ (true positive + false negative)

^**γ**^Specificity = true negative/ (true negative + false positive)

^δ^Positive predictive value = true positive/ (true positive + false positive)

^ε^F1 score = 2 ((sensitivity*PPV)/ (sensitivity + PPV))

**Table 3:** Performance of the image type query in identifying specific image types in the 361-patient cohort^ζ^

Image type				
Bone scan			Gold standard manual abstraction	
			Bone scan	Not bone scan
	Automated Lucene tool result	Bone scan	23	0
		Not bone scan	0	2988
	Sensitivity (95% CI)	Specificity (95% CI)	PPV (95% CI)	F1 score
	100% (85–100%)	100% (100–100%)	100% (85–100%)	1.00
Chest X-ray			Gold standard manual abstraction	
			Chest X-ray	Not chest X-ray
	Automated Lucene tool result	Chest X-ray	1039	0
		Not chest X-ray	281	1691
	Sensitivity (95% CI)	Specificity (95% CI)	PPV (95% CI)	F1 score
	79% (76–81%)	100% (100–100%)	100% (100–100%)	0.88
CT chest			Gold standard manual abstraction	
			CT chest	Not CT chest
	Automated Lucene tool result	CT chest	841	0
		Not CT chest	61	2109
	Sensitivity (95% CI)	Specificity (95% CI)	PPV (95% CI)	F1 score
	93% (91–95%)	100% (100–100%)	100% (100–100%)	0.97
CT abdomen/pelvis			Gold standard manual abstraction	
			CT abdomen/pelvis	Not CT abdomen/pelvis
	Automated lucene tool result	CT abdomen/pelvis	63	0
		Not CT abdomen/pelvis	64	2884
	Sensitivity (95% CI)	Specificity (95% CI)	PPV (95% CI)	F1 score
	50% (41–59%)	100% (100–100%)	100% (94–100%)	0.66
CT chest/abdomen/pelvis			Gold standard manual abstraction	
			CT Chest/abdomen/pelvis	Not CT Chest/abdomen/pelvis
	Automated Lucene tool result	CT Chest/abdomen/pelvis	116	0
		Not CT Chest/abdomen/pelvis	7	2888
	Sensitivity (95% CI)	Specificity (95% CI)	PPV (95% CI)	F1 score
	94% (89–98%)	100% (100–100%)	100% (97–100%)	0.97
CT head			Gold standard manual abstraction	
			CT head	Not CT head
	Automated Lucene tool result	CT head	131	0
		Not CT head	18	2862
	Sensitivity (95% CI)	Specificity (95% CI)	PPV (95% CI)	F1 score
	88% (82–93%)	100% (100–100%)	100% (97–100%)	0.94
MR body			Gold standard manual abstraction	
			MR body	Not MR body
	Automated Lucene tool result	MR body	28	0
		Not MR body	30	2953
	Sensitivity (95% CI)	Specificity (95% CI)	PPV (95% CI)	F1 score
	48% (35–62%)	100% (100–100%)	100% (88–100%)	0.65
MR brain			Gold standard manual abstraction	
			MR brain	Not MR brain
	Automated Lucene tool result	MR brain	41	0
		Not MR brain	1	2969
	Sensitivity (95% CI)	Specificity (95% CI)	PPV (95% CI)	F1 score
	98% (87–100%)	100% (100–100%)	100% (91–100%)	0.99
PET			Gold standard manual abstraction	
			PET	Not PET
	Automated Lucene tool result	PET	266	0
		Not PET	1	2744
	Sensitivity (95% CI)	Specificity (95% CI)	PPV (95% CI)	F1 score
	100% (98–100%)	100% (100–100%)	100% (99–100%)	1.00

^ζ^This 361-patient cohort consists of 3011 manually abstracted reports

Comparison of the timing results between manual abstraction and semi-automated abstraction using Lucene for the image type query are presented in Table 4. Given the comparatively lower performance of all computerized queries with the exception of image type, we restricted our analysis of timing of the semi-automated methods to this query alone. Using the image type query, we were able to reduce the abstractor time spent per patient by 68%, from a median of 21.5 min/patient (IQR 16.0) to 6.9 min/patient (IQR 9.5) (p = 0.0024). Time spent per report was reduced by 50% from a median of 60.0 s (IQR 90.0 s) to 30.0 s (IQR 80.0 s) (p < 0.0005). However, there was a significantly smaller number of reports per patient in those examined with the semi-automatic technique v. manual abstraction technique (mean ± SD, 9.96 ± 9.41 v. 12.75 ± 10.50 (p = 0.04)) by virtue of the goals of the query to reduce the number of irrelevant reports that required review.

**Table 4:** Timing for manual and semi-automated chart abstraction^ζ^

Timing metric	Manual	Semi-automated	p-value	% Reduction in time
Total number of reports	204	239		
Minutes/patient (median, (IQR))	21.5 (16.0)	6.9 (9.5)	0.0024	68
Seconds/patient report (median, (IQR))	60.0 (90.0)	30.0 (80.0)	< 0.0005	50
Reports/patient (mean, (SD))	12.75 (10.50)	9.96 (9.41)	0.0398	

^**ζ**^The semi-automated chart abstraction was performed using the image-type query

## Discussion

Our goal was to develop a methodology that would allow us to efficiently gather clinically accurate information from unstructured radiology text reports housed in the VA CDW database. Ultimately, we would like to use this information to help inform post lung cancer treatment surveillance guidelines. To do this we have developed a semi-automated process to chart abstraction using computerized queries to identify image types, images ordered explicitly for surveillance, and images that demonstrated findings suspicious or definitive for lung cancer recurrence, metastasis, or second primary lung cancer.

An ideal method would be evidenced by a query which yields a high PPV, reliably only retrieving relevant image reports. This reduces the time spent reviewing and ultimately excluding irrelevant reports allowing the abstractor to concentrate their efforts on annotating only those reports relevant to the outcome of interest. This must be balanced with high test sensitivity as well. A highly sensitive query improves efficiency by decreasing the time a manual abstractor would spend searching for unidentified results. Thus efficient, high performing queries with high PPV and sensitivity provide an important pre-processing step to manual abstraction. They retrieve a high proportion of the relevant image reports while excluding irrelevant reports and thus reducing time spent on unnecessary chart review.

In this study, we hypothesized that the computerized queries on their own would perform this pre-processing step sufficiently well to improve efficiency but would likely be unable to yield high enough accuracy when used alone in the absence of manual validation. The queries would predictably have limitations in their ability to categorize the image characteristics (image indication and image findings) on their own. Thus, using a semi-automated approach, would allow the use of computerized queries to improve time efficiency by decreasing the number of irrelevant reports reviewed manually and by retrieving a significant number of the relevant reports while also decreasing any limitations in accuracy that would arise when using the queries on their own.

Overall, the image type query was the best performing query with sensitivity 85%, PPV 95%, and F1 Score 0.90. By contrast, computerized queries to identify CT abdomen/pelvis and MRI body were only able to reliably code these image types approximately 50% of the time. It is not clear as to why these particular image types had low sensitivity. However, given the overall success of this query, we subsequently attempted to determine if use of this query did improve efficiency of abstraction. The use of this query did reduce the amount of time necessary for abstraction. There was a 68% reduction in abstraction time per patient and 50% reduction in abstraction time per report. It is key to note that there were, on average 3 less reports to review per patient in the group of reports abstracted using the semi-automated method vs. manual abstraction. However, given the significant reduction in time from 21.5 min/patient using the manual method to 6.9 min per patient using the semi-automated method, it is unlikely that a difference of approximately 3 reports per patient could account for this difference in its entirety. Indeed, the objective and main benefit of the semi-automated method is to do just this, reduce the work associated with abstraction of irrelevant data and in fact, when normalized to time spent per image report, the reduction in time associated with the semi-automated abstraction persisted.

The performance of the surveillance indication query was intermediate with sensitivity 72%, PPV 70%, and F1 Score 0.71. The computerized query for image findings to characterize cancer recurrence demonstrated the worst test performance with PPV of 25% (suspicious) and 23% (recurrence). However, the query did demonstrate an intermediate sensitivity of 75% (suspicious) and 85% (recurrence). The F1 scores were 0.37 (suspicious) and 0.36 (recurrence). The PPV of the image findings queries was much lower than expected. The remaining results, sensitivity of the image findings queries and the PPV and sensitivity of the image indication queries were satisfactory as expected. However, given that the best queries have both high PPV and high sensitivity, this finding continues to emphasize that the queries benefit from manual abstraction to improve upon accuracy.

Our results demonstrate the limitations of using computerized queries alone to fully characterize imaging reports from unstructured data in the complicated clinical setting of radiology reports for lung cancer patients following treatment. The VISA search tool works well for identifying relevant image types which then defines the point at which a trained abstractor must be utilized to accurately complete the abstraction process. The primary benefit of the VISA tool/queries is that it does improve efficiency and decrease the amount of time for abstraction given the high PPV and sensitivity of the image type query and its ability to include only relevant reports. This study helps to define the limitations of the VISA tool to maximize its benefit (improved efficiency) while limiting the harms of data inaccuracy.

This study adds to growing literature examining the use of natural language processing and machine learning to explore surveillance radiology patterns and clinical outcomes following treatment for lung cancer. Wadia et al., compared a rule-based natural language processing strategy and combination natural language processing/manual abstraction to identify CT reports with findings suspicious for a new lung cancer diagnosis. Though slightly different from our aim (to examine multiple imaging types for lung cancer recurrence), they also found that a combination of natural language processing and manual abstraction was the most successful. The sensitivity and PPV for natural lanague processing alone was 77% and 88%, respectively. With a combination of natural language processing and manual abstraction the sensitivity was 92% and the PPV was 87% [23]. Hunter et al. also analyzed the ability to detect “concerning” and “reassuring” lung nodules on CT using different machine learning algorithms (Logistic regression, XG boost, Naïve Bayes, Random forest, Linear support vector machine). They initially identified CT scans with lung nodules using a validated rule-based natural language approach. Overall the F1 scores ranged from 0.71 to 0.89, Sensitivity ranged from 0.70–0.90, and PPV ranged from 0.64–0.89. The best performing algorithm was linear support vector machine with F1 0.89, sensitivity 0.90, and PPV 0.88 for predicting concerning lung nodules [24]. It is important to note, the machine learning technique was used after a pre-processing step using a rule-based algorithm to identify CT reports that actually had nodules. Our study also used a computerized pre-processing step to identify relevant image reports to focus on, however, we attempted to differentiate between multiple different image types, which results in added complexity.

Other studies in the field of natural language processing have also sought to find the most appropriate way to extract relevant information from unstructured data by using more automated approaches. For example, Steinkamp et al. [31] like our group, sought to extract structured data—findings, recommendations, clinical history indications, etc.—from abdominopelvic radiology reports. They however, used a technique that relied solely on an automated machine learning process by identifying both anchor words associated with the sought-after data and surrounding modifier terms to help contextualize and identify further information. They had very good results with sensitivity of 88.6%, positive predictive values of 93.5% and F1 score of 91.0%. This demonstrates as we predicted that simply using an automated method of identifying anchor words associated with the sought-after data is not sufficient, one must further contextualize the anchor words within a query with either human input (as in our study) or further machine learning as demonstrated by Steinkamp et al. [31].

Another notable study by Casey et al., reviewed techniques to extract information from radiology reports using computerized methods. Per Casey et al. [32], our approach to extract information would be considered “rule-based” as we used specific words or terms that presented frequently to search through the text. Though as our approach is semi-automated (utilizing human review as well), this label does not completely describe our technique. Per Casey et al. since 2015 the number of studies utilizing a “rule-based” approach is approximately 10–15 annually,with the number of studies incorporating further machine learning increasing over time. Though given the aforementioned challenges to automated processes, there is still work to do prior to those techniques being fully adapted for clinical research. For example, machine learning or deep learning techniques require large amounts of data for training, such that they remain inadequate/inaccurate for small sample sizes. Thus utilizing a semi-automated approach, we are able to circumvent this potential problem [32].

There are notable limitations to this study. The computerized queries may have contained overly inclusive language resulting in inclusion of irrelevant studies when tested on the larger gold standard cohort. This was not an issue for the image type query as it was able to very efficiently retrieve relevant image reports. It is likely that the language used to identify an image type is more standardized than that needed to identify image indications or findings thus contributing to the observed superior accuracy for this query.

This may also have introduced potential uncertainty in the actual number of false positives and true negatives captured with our image type query. Image type was manually abstracted in 3011 reports. In addition to this, 180 reports were coded as null by our abstractor. The null reports commonly represented image types that were irrelevant for lung cancer surveillance (i.e., a foot x-ray or pelvic ultrasound). These null reports were only included when examining the image type query’s yield. The image type query, designed to retrieve any relevant image, was unable to retrieve reports grouped by type. (i.e. CT chest and CT head were returned together). Thus, we were not able to determine the exact number of false positives or true negatives for each specific type, as the image type query did not discern image types. 

Despite these limitations, we belive the VISA tool and the semi-automated abstraction method is still of great benefit. The VISA tool does not require a predictable format for provider progress notes, radiology text notes and other text-based data. This is critical given the non-standard way in which radiology reports are dictated by radiologists even within the same healthcare system. In this technique, key words are highlighted allowing the abstractor to rapidly identify relevant sections and skip over unnecessary sections or in this case, to skip entire radiology reports that are not relevant to the study. This also allows for greater efficiency in chart abstraction. In our prior unpublished lung nodule pilot, a single expert reviewer was able to annotate 1292 reports in 154 h reviewing approximately 168 reports per day (average rate of 2.5 min per chart). In other published studies, the VISA tool was able to identify discussions about initiation of dialysis treatment from clinical provider notes for 19,165 individuals and able to successfully identify adverse childhood events from 44.7 million clinical notes among 243,973 Gulf War Veterans(6, 27). It is clear that the use of queries in our Lucene-based VISA tool is helpful. Our semi-automated method utilizes the efficiency of this technique but improves upon concerns about inaccuracies. Thus, this technique shows significant potential for use in large data studies requiring electronic health record review.

## Conclusions

The image type query acts as a pre-processing tool for manual abstraction, retrieving a significant proportion of mostly relevant images. However, the intermediate to poor performance of the image indication and findings queries indicates the necessity of semi-automated abstraction for appropriate report characterization and is likely necessary for more detailed clinical data from unstructured sources. The semi-automated abstraction method using the image type query will thus be a useful tool in future studies.

## Supplementary Information

**Additional file 1:** Appendices A–C. **Appendix A:** ICD-9 and CPT/HCPCS codes. **Appendix B:** Consort diagram. **Appendix C:** Search queries.

## Data Availability

The data that support the findings of this study are available from The Veterans Health Association corporate data warehouse and the radiographic index that our research group created but restrictions apply to the availability of these data, which were used under license for the current study, and so are not publicly available. Data are however available from the authors upon reasonable request and with permission of the Veterans Health Association.

## Abbreviations

PPV: Positive predictive value
IQR: Interquartile range
NCCN: National Comprehensive Cancer Network
SEER: Surveillance, Epidemiology and End Results Program
VACCR: Veterans Affairs Central Cancer Registry
VHA: Veterans Health Administration
CDW: Corporate Data Warehouse
VISA: Veterans Indexed Search for Analytics
NSCLC: Non-small cell lung cancer
VA: Veterans Administration
ICD: International Classification of Diseases
CPT: Current Procedural Terminology
HCPCS: Healthcare Common Procedure Coding System
TIU: Text Integration Utility
