# Supplementary material for: Performance of a rule-based semi-automated method to optimize chart abstraction for surveillance imaging among patients treated for non-small cell lung cancer
Source: BMC Med Inform Decis Mak. 2022 Jun 3;22:148. doi: 10.1186/s12911-022-01863-0 (PMC9166440; doi:10.1186/s12911-022-01863-0)
Supplement: Supplementary file 1 — Additional file 1: Appendices A–C. Appendix A: ICD-9 and CPT/HCPCS codes. Appendix B: Consort diagram. Appendix C: Search queries. [file 12911_2022_1863_MOESM1_ESM.docx]

**Appendix A: CPT/HCPCS and ICD-9 codes**

| Lung cancer diagnoses | ICD-9 Codes | |
| --- | --- | --- |
| Malignant neoplasm of Trachea | 162.2 | |
| Malignant neoplasm of Upper lobe, bronchus or lung | 162.3 | |
| Malignant neoplasm of Middle lobe, bronchus or lung | 162.4 | |
| Malignant neoplasm of Lower lobe, bronchus or lung | 162.5 | |
| Malignant neoplasm of Other parts of bronchus or lung (includes malignant neoplasm of contiguous or overlapping sites of bronchus or lung whose point of origin cannot be determined) | 162.8 | |
| Malignant neoplasm of Bronchus and lung, unspecified | 162.9 | |
| Surgical | Description | CPT/HCPCS |
| Lung Resection |  |  |
|  | Surgical Resection (All) | 32440, 32442, 32445, 32480, 32484, 32486, 32488, 32500, 32520, 31766, 32525, 32657, 32663, 32522, 32663 |
|  | Lobectomy | 32480, 32486, 32488, 32503, 32504, 32663 |
|  | Wedge Resection | 32505, 32506, 32608, 32666, 32667 |
|  | Segmentectomy | 32484, 32500, 32657 |
|  | Pneumonectomy | 32440, 32445 |
|  | Carinal pneumonectomy | 32442 |
|  | Bilobectomy | 32482 |
|  | VATS Lung Resection | 32663, 32666, 32667, 32669, 32670, 32671 |
| Mediastinal Surgery |  |  |
|  | Mediastinoscopy | 39400 |
|  | Mediastinotomy | 39000, 39010 |
|  | Nodal biopsy | 32605, 32606 |
| Lymphadenectomy |  |  |
|  | Lymphadenectomy | 38746 |
| Other Surgery |  |  |
|  | Thoracentesis | 32421 |
|  | Tunneled pleural catheter | 32550, 32552 |
|  | Tube thoracostomy | 32422, 32551 |
|  | Pleurodesis | 32560, 32561, 32562 |
|  | VATS drainage of pleural effusion |  |
|  | VATS pleurodesis | 32650 |
|  | VATS decortication | 32651, 32652 |
|  | VATS pericardial window | 32659 |
| Systemic Therapy |  |  |
|  | Radiation therapy | 31643, 77332, 77404, 77418, 77522, 77799, C1795, C1806, 77300, 77333, 77406, 77419, 77523, C1716, C1796, C2616, 77301, 77334, 77407, 77420, 77525, C1717, C1797, G0126, 77305, 77336, 77408, 77425, 77750, C1718, C1798, G0173, 77310, 77370, 77409, 77427, 77761, C1719, C1799, 77315, 77380, 77411 77430, 77762, C1720, C1800, 77321, 77381, 77412, 77431, 77763 C1790, C1801, 77326, 77399, 77413, 77432, 77781, C1791, C1802 77327, 77401, 77414, 77470, 77782, C1792, C1803, 77328, 77402 77416, 77499, 77783, C1793, C1804, 77331, 77403, 77417, 77520 77784, C1794, C1805, |
|  | Chemotherapy | 95549, 96425, J8610, J9091, J9190, J9350, Q0127, 96400, 96440, J899, J9092, J9201, J9360, Q0128, 96404, 96445, J9000, J9093, J9206, J9370, Q0129, 96406, 96450, J9001, J9094, J9208, J9375, S0178, 96410, 96542, J9010, J9095, J9230, J9380, S018296412, 96545, J9045, J9096, J9250, J9390, S9329, 96414, C9017, J9060, J9097, J9260, J9999, S9330, 96420, J0182, J9062, J9170, J9265, Q0083, S9331, 96420, J8510, J9070, J9180, J9280, Q0084, 96422, J8530, J9080, J9181, J9290, Q0085, 96423, J8560, J9090, J9182, J9291, Q0125 |
| Other Procedures: |  |  |
|  | Endoscopic ultrasound | 43232, 43242, 43259, 76975 |
|  | Endobronchial ultrasound | 31620 |
|  | Lymph node biopsy | 32400, 32402, 32405 |
|  | Bronchoscopy | 31622, 31623, 31624, 31225, 31627, 31628, 31629, 31630, 31631, 31632, 31633, 31636, 31637, 31638, 31640, 31641, 31645, 31646 |

**Appendix B: Consort diagram**

Non-Small Cell Lung Cancer patients diagnosed between 2008-2016 VA/CDW database

(n=185,112)

Patients with any relevant procedure/treatment

(n=56,439)

**Excluded:**

1. Other Procedure/No Treatment

(n=129,673)

Patients with any relevant procedure/treatment within -1 to 6 months from index diagnosis

(n=38,564)

**Excluded:**

1. Procedure before a month and after 6 months of index diagnosis

(n=17,875)

Final Cohort

**(n=17,472)**

**Excluded:**

1. Patient who died within 6 months of diagnosis (n=14,092)
2. Patients That Had Previous Cancer in Required interval (NOT within 5 previous years of index dx (n=5,182)
3. Patients with stage IV cancer (n=1,818)

**Appendix C: Search Queries**

*Boolean Image Type Query:* The default operator between phrase queries is OR (e.g the VISA tool will retrieve image types with either “MRI Brain” OR “CT head” within the body of the report)

“MRI Brain" "CT head" "CT chest" "CT thorax"  "chest x  ray" "CXR" "Positron" "PET" "Bone Scan" "CT chest abdomen pelvis" "CT abdomen pelvis" "CT Brain" "Magnetic Resonance" "Computerized Tomography" "MRI abdomen" "MRI Liver" "CT ABD PELV" "PA  Lateral" "exam of chest" "examination of the chest" "chest PA & LAT" "PA and lateral" "CT images through the chest" "MRI of the brain"  "CT scan of the chest" "MRI of the abdomen" "CT examination  of the abdomen and pelvis" "single view of the chest" "four view" "sagittal T1" "brain" "2 view" "CT of the chest" "Comput tomography" "Thoracic CT"  "CT lung" "CT scan lung" "head MRI" "MRI head" "head CT" "thorax CT" "liver MRI" "abdomen MRI" "Ct images of the chest" "Chest 2" "two view" "single portable view" "portable chest" "4 view" "portable AP chest" "images of the chest" "images through the thorax" "lateral chest" "PA & LAT" "CT of the thorax" "AP portable" "CT scan of abdomen and pelvis" "chest single view" "portable AP"  "chest single frontal projection" "Chest CT" "CT the chest" "1 view frontal" "chest wo" "chest radiograph" "radiograph of chest" "chest xr" "chest xray" “somatostatin receptor scintigraphy” “octreotide scan”

---------------------------------------------------------------------------------------------------------------------

*Boolean Surveillance Query:* This query was built using reports annotated as surveillance screening indication and used in conjunction with the parent query representing 1137 patients and 2550 reports. The default operator between phrase queries is OR

Surveillance, “history of lung cancer”, “history lung cancer”, “hx of lung cancer” “hx lung cancer” “history of lung ca”, “history lung ca” “hx of lung ca” “hx lung ca” “history of lung carcinoma” “indication lung cancer” indication lung ca” indication lung carcinoma” follow up lung cancer” “followup lung cancer” “f/u lung cancer”” follow up lung carcinoma” “f/u lung ca” “followup lung carcinoma” f/u lung carcinoma” “r/o mets” restaging” screening” “r/o recurrence” “indication cancer” clinical lung cancer” “history adenocarcinoma” “history of adenocarcinoma” “history of nsclc” “hx nsclc” “hx of nsclc” “hx scc” “hx of scc” “history of squamous cell” “hx of squamous cell” “cancer follow up “ “cancer followup” “cancer f/u” “lung cancer s/p” “lung cancer status post” “adenocarcinoma s/p” “adenocarcinoma status post” “history of non small cell” “hx of non small cell” “nsclc s/p” “scc s/p” “scc status post” “squamous cell carcinoma s/p” “squamous cell carcinoma status post” “s/p lobectomy” “post lobectomy” “ s/p wedge” “post wedge” “s/p chemo” “post chemo” “post chemotherapy” “s/p radiation” “status post radiation” “s/p SBRT”” “r/o brain met” “remote lung ca” “h/o lung cancer” “fu lung ca” “lung cancer sp” “history of cancer” evaluate for tumor recurrence” “ evaluate for metastatic” “carcinoma resection” “r/o recurrence” “evaluate for lung cancer” “indication non small cell” “lobectomy for carcinoma” “assess for recurrence” “history of lung nodule” “interval followup” “interval follow up” “interval f/u” “lung ca s/p” “post radiation” “history of carcinoid” “nsclc f/u” “staging” “evaluate for recurrent” “ct followup” “reasn for exam lung cancer” “assessment for malignant” reason for study eval” for lung cancer” “followup of lung cancer” “h/o ca lung” “nodule followup” “eval for lung cancer recurrence” “lung malignancy status post” “r/o metastasis” “h/o ca of lung” “h/o lung ca” “reason for study lung cancer” “h/o squamous cell” “h/o NSCLC” “eval for recurrence”

---------------------------------------------------------------------------------------------------------------------

*Span Suspicious Query:* This query was built using reports annotated as suspicious in conjunction with the parent query representing 462 patients, 711 reports. This query retrieves reports with the words signifying suspicious AND cancer within 10 words of each other in the document. If the words “not” or “no” are found between within the 10 words, the report is excluded. Each line is linked by the operator OR.

Near: suspicious mass| slope: 10| any order|not: not no

Near: suspicious progression| slope: 10| any order|not: not no

Near: suspicious nodule| slope: 10| any order|not: not no

Near: suspicious metastases| slope: 10| any order|not: not no

Near: suspicious metastasis| slope: 10| any order|not: not no

Near: suspicious metastatic| slope: 10| any order|not: not no

Near: suspicious recurrence| slope: 10| any order|not: not no

Near: suspicious malignancy| slope: 10| any order|not: not no

Near: suspicious neoplasm| slope: 10| any order|not: not no

Near: suspicious neoplastic| slope: 10| any order|not: not no

Near: suspicious cancer| slope: 10| any order|not: not no

Near: worrisome mass| slope: 10| any order|not: not no

Near: worrisome progression| slope: 10| any order|not: not no

Near: worrisome nodule| slope: 10| any order|not: not no

Near: worrisome metastases| slope: 10| any order|not: not no

Near: worrisome metastasis| slope: 10| any order|not: not no

Near: worrisome metastatic| slope: 10| any order|not: not no

Near: worrisome recurrence| slope: 10| any order|not: not no

Near: worrisome malignancy| slope: 10| any order|not: not no

Near: worrisome neoplasm| slope: 10| any order|not: not no

Near: worrisome neoplastic| slope: 10| any order|not: not no

Near: worrisome cancer| slope: 10| any order|not: not no

Near: concerning mass| slope: 10| any order|not: not no

Near: concerning progression| slope: 10| any order|not: not no

Near: concerning nodule| slope: 10| any order|not: not no

Near: concerning metastases| slope: 10| any order|not: not no

Near: concerning metastasis| slope: 10| any order|not: not no

Near: concerning metastatic| slope: 10| any order|not: not no

Near: concerning recurrence| slope: 10| any order|not: not no

Near: concerning recurrent| slope: 10| any order|not: not no

Near: concerning malignancy| slope: 10| any order|not: not no

Near: concerning neoplasm| slope: 10| any order|not: not no

Near: concerning neoplastic| slope: 10| any order|not: not no

Near: concerning cancer| slope: 10| any order|not: not no

Near: probable mass| slope: 10| any order|not: not no

Near: probable progression| slope: 10| any order|not: not no

Near: probable nodule| slope: 10| any order|not: not no

Near: probable metastases| slope: 10| any order|not: not no

Near: probable metastasis| slope: 10| any order|not: not no

Near: probable metastatic| slope: 10| any order|not: not no

Near: probable recurrence| slope: 10| any order|not: not no

Near: probable malignancy| slope: 10| any order|not: not no

Near: probable neoplasm| slope: 10| any order|not: not no

Near: probable neoplastic| slope: 10| any order|not: not no

Near: probable cancer| slope: 10| any order|not: not no

Near: possible mass| slope: 10| any order|not: not no

Near: possible progression| slope: 10| any order|not: not no

Near: possible nodule| slope: 10| any order|not: not no

Near: possible metastases| slope: 10| any order|not: not no

Near: possible metastasis| slope: 10| any order|not: not no

Near: possible metastatic| slope: 10| any order|not: not no

Near: possible recurrence| slope: 10| any order|not: not no

Near: possible malignancy| slope: 10| any order|not: not no

Near: possible neoplasm| slope: 10| any order|not: not no

Near: possible cancer| slope: 10| any order|not: not no

Near: suspicious mass| slope: 10| any order|not: not no

Near: “could represent” progression| slope: 10| any order|not: not no

Near: “could represent” nodule| slope: 10| any order|not: not no

Near: “could represent” metastases| slope: 10| any order|not: not no

Near: “could represent” metastasis| slope: 10| any order|not: not no

Near: “could represent” metastatic| slope: 10| any order|not: not no

Near: “could represent” recurrence| slope: 10| any order|not: not no

Near: “could represent” malignancy| slope: 10| any order|not: not no

Near: “could represent” neoplasm| slope: 10| any order|not: not no

Near: “could represent” neoplastic| slope: 10| any order|not: not no

Near: “could represent” cancer| slope: 10| any order|not: not no

Near: “may represent” progression| slope: 10| any order|not: not no

Near: “may represent” nodule| slope: 10| any order|not: not no

Near: “may represent” metastases| slope: 10| any order|not: not no

Near: “may represent” metastasis| slope: 10| any order|not: not no

Near: “may represent” metastatic| slope: 10| any order|not: not no

Near: “may represent” recurrence| slope: 10| any order|not: not no

Near: “may represent” malignancy| slope: 10| any order|not: not no

Near: “may represent” neoplasm| slope: 10| any order|not: not no

Near: “may represent” neoplastic| slope: 10| any order|not: not no

Near: “may represent” cancer| slope: 10| any order|not: not no

Near: suggestive mass| slope: 10| any order|not: not no

Near: suggestive progression| slope: 10| any order|not: not no

Near: suggestive nodule| slope: 10| any order|not: not no

Near: suggestive metastases| slope: 10| any order|not: not no

Near: suggestive metastasis| slope: 10| any order|not: not no

Near: suggestive metastatic| slope: 10| any order|not: not no

Near: suggestive recurrence| slope: 10| any order|not: not no

Near: suggestive malignancy| slope: 10| any order|not: not no

Near: suggestive neoplasm| slope: 10| any order|not: not no

Near: suggestive neoplastic| slope: 10| any order|not: not no

Near: suggestive cancer| slope: 10| any order|not: not no

Near: questionable mass| slope: 10| any order|not: not no

Near: questionable progression| slope: 10| any order|not: not no

Near: questionable nodule| slope: 10| any order|not: not no

Near: questionable metastases| slope: 10| any order|not: not no

Near: questionable metastasis| slope: 10| any order|not: not no

Near: questionable metastatic| slope: 10| any order|not: not no

Near: questionable recurrence| slope: 10| any order|not: not no

Near: questionable malignancy| slope: 10| any order|not: not no

Near: questionable neoplasm| slope: 10| any order|not: not no

Near: questionable neoplastic| slope: 10| any order|not: not no

Near: questionable cancer| slope: 10| any order|not: not no

Near: differential mass| slope: 10| any order|not: not no

Near: differential progression| slope: 10| any order|not: not no

Near: differential nodule| slope: 10| any order|not: not no

Near: differential metastases| slope: 10| any order|not: not no

Near: differential metastasis| slope: 10| any order|not: not no

Near: differential metastatic| slope: 10| any order|not: not no

Near: differential recurrence| slope: 10| any order|not: not no

Near: differential malignancy| slope: 10| any order|not: not no

Near: differential neoplasm| slope: 10| any order|not: not no

Near: differential neoplastic| slope: 10| any order|not: not no

Near: differential cancer| slope: 10| any order|not: not no

Near: “cannot be excluded” neoplasm| slope: 10| any order|not: not no

Near: “cannot be excluded” recurrence| slope: 10| any order|not: not no

Near: “cannot be excluded” malignancy| slope: 10| any order|not: not no

Near: “cannot be excluded” cancer| slope: 10| any order|not: not no

Near: consideration metastatic | slope: 10| any order|not: not no

Near: consideration recurrence| slope: 10| any order|not: not no

Near: consideration malignancy| slope: 10| any order|not: not no

Near: slight “interval increase” | slope: 10| any order|not: not no

Near: mild “interval increase” | slope: 10| any order|not: not no

Near: “likely represents” recurrent| slope: 10| any order|not: not no

Near: suspicious lymphadenopathy| slope: 10| any order|not: not no

Near: cannot exclude recurrent| slope: 10| any order|not: not no

Near: neoplastic cannot exclude slope: 10| any order|not: not no

Near: concerning recurrence| slope: 10| any order|not: not no

Near: mild hypermetabolic| slope: 10| any order|not: not no

Near: mildly hypermetabolic| slope: 10| any order|not: not no

Near: focal thickening| slope: 10| any order|not: not no

Near: mild thickening| slope: 10| any order|not: not no

Near: focal enhancement| slope: 10| any order|not: not no

Near: suspicion metastatic| slope: 10| any order|not: not no

Near: differential tumor| slope: 10| any order|not: not no

Near: new nodule| slope: 10| any order|not: not no

Near: concerning “nodal involvement| slope: 10| any order|not: not no

Near: mass like opacity| slope: 10| any order|not: not no

Near: nodular thickening| slope: 10| any order|not: not no

Near: enlarged node| slope: 10| any order|not: not no

Near: increased node| slope: 10| any order|not: not no

Near: mass grown| slope: 10| any order|not: not no

Near: nodule increased| slope: 10| any order|not: not no

---------------------------------------------------------------------------------------------------------------------

*Span Recurrent Query:* This query was built using reports annotated as recurrent in conjunction with the parent query representing 230 patients and 241 reports. This query retrieves reports with the words signifying definite AND cancer within 10 words of each other in the document. If the words “not” or “no” are found between within the 10 words, the report is excluded. Each line is linked by the operator OR.

Near: “consistent with” recurrence| slope: 10| any order|not: not no

Near: “compatible with” recurrence| slope: 10| any order|not: not no

Near: represents recurrence| slope: 10| any order|not: not no

Near: demonstrates recurrence| slope: 10| any order|not: not no

Near: “highly suspicious” recurrence| slope: 10| any order|not: not no

Near: progression recurrence| slope: 10| any order|not: not no

Near: “consistent with” metastasis| slope: 10| any order|not: not no

Near: “compatible with” metastasis| slope: 10| any order|not: not no

Near: represents metastasis| slope: 10| any order|not: not no

Near: demonstrates metastasis| slope: 10| any order|not: not no

Near: “highly suspicious” metastasis| slope: 10| any order|not: not no

Near: progression metastasis| slope: 10| any order|not: not no

Near: “consistent with” metastatic| slope: 10| any order|not: not no

Near: “compatible with” metastatic slope: 10| any order|not: not no

Near: represents metastatic| slope: 10| any order|not: not no

Near: demonstrates metastatic| slope: 10| any order|not: not no

Near: “highly suspicious” metastatic| slope: 10| any order|not: not no

Near: progression metastatic| slope: 10| any order|not: not no

Near: “consistent with” malignancy| slope: 10| any order|not: not no

Near: “compatible with” malignancy| slope: 10| any order|not: not no

Near: represents malignancy| slope: 10| any order|not: not no

Near: demonstrates malignancy| slope: 10| any order|not: not no

Near: “highly suspicious” malignancy| slope: 10| any order|not: not no

Near: progression malignancy| slope: 10| any order|not: not no

Near: “consistent with” neoplasm| slope: 10| any order|not: not no

Near: “compatible with” neoplasm| slope: 10| any order|not: not no

Near: represents neoplasm| slope: 10| any order|not: not no

Near: demonstrates neoplasm| slope: 10| any order|not: not no

Near: “highly suspicious” neoplasm| slope: 10| any order|not: not no

Near: progression neoplasm| slope: 10| any order|not: not no

Near: “consistent with” neoplastic| slope: 10| any order|not: not no

Near: “compatible with” neoplastic| slope: 10| any order|not: not no

Near: represents neoplastic| slope: 10| any order|not: not no

Near: demonstrates neoplastic| slope: 10| any order|not: not no

Near: “highly suspicious” neoplastic| slope: 10| any order|not: not no

Near: progression neoplastic| slope: 10| any order|not: not no

Near: mass recurrence| slope: 10| any order|not: not no

Near: mass metastasis| slope: 10| any order|not: not no

Near: mass metastatic| slope: 10| any order|not: not no

Near: mass malignancy| slope: 10| any order|not: not no

Near: mass neoplasm| slope: 10| any order|not: not no

Near: mass neoplastic| slope: 10| any order|not: not no

Near: lymph node metastasis| slope: 10| any order|not: not no

Near: liver metastasis| slope: 10| any order|not: not no

Near: brain metastasis| slope: 10| any order|not: not no

Near: adrenal metastasis| slope: 10| any order|not: not no

Near: progression disease| slope: 10| any order|not: not no

Near: “lymph node” recurrence| slope: 10| any order|not: not no

Near: characteristic malignant| slope: 10| any order|not: not no

Near: characteristic malignancy| slope: 10| any order|not: not no

Near: characteristic neoplasm| slope: 10| any order|not: not no

Near: characteristic recurrence| slope: 10| any order|not: not no

Near: characteristic metastatic| slope: 10| any order|not: not no

Near: characteristic metastasis| slope: 10| any order|not: not no

Near: presume metastases| slope: 10| any order|not: not no

Near: presumed malignant| slope: 10| any order|not: not no

Near: presumed recurrence| slope: 10| any order|not: not no

Near: “associated with” recurrence| slope: 10| any order|not: not no

Near: “consistent with” tumor| slope: 10| any order|not: not no

Near: “compatible with” tumor| slope: 10| any order|not: not no

Near: represents tumor| slope: 10| any order|not: not no

Near: demonstrates tumor| slope: 10| any order|not: not no

Near: “highly suspicious” tumor| slope: 10| any order|not: not no

Near: progression tumor| slope: 10| any order|not: not no

Near: pathologic fracture| slope: 10| any order|not: not no

Near: growing mass| slope: 10| any order|not: not no

Near: represents carcinoma| slope: 10| any order|not: not no

Near: “compatible with” metastases| slope: 10| any order|not: not no

Near: “consistent with” metastases| slope: 10| any order|not: not no

Near: represents metastases| slope: 10| any order|not: not no

Near: “highly suspicious” metastases| slope: 10| any order|not: not no

Near: demonstrates metastases| slope: 10| any order|not: not no

Near: progression metastases| slope: 10| any order|not: not no

Near: increased mass| slope: 10| any order|not: not no

Near: cavitary mass| slope: 10| any order|not: not no

Near: necrotic mass| slope: 10| any order|not: not no

Near: enlarging metastasis| slope: 10| any order|not: not no

Near: metastatic lesions| slope: 10| any order|not: not no

Near: lytic lesions| slope: 10| any order|not: not no

Near: progression mass| slope: 10| any order|not: not no
